# Biomechanical evaluation of oblique lateral interbody fusion with various fixation methods for degenerative lumbar scoliosis: a finite element analysis considering different bone densities

**DOI:** 10.3389/fbioe.2025.1562268

**Published:** 2025-05-08

**Authors:** Wei Guo, Zemin Wang, Meina Song, Wei Yang, Honglai Zhang, Wanzhong Yang, Shiyong Wang, Rong Ma, Zhaohui Ge

**Affiliations:** ^1^ Department of Orthopedic, General Hospital of Ningxia Medical University, Yinchuan, China; ^2^ First Clinical Medical College, Ningxia Medical University, Yinchuan, China; ^3^ Department of Radiology, Baoji Traditional Chinese Medicine Hospital, Baoji, China

**Keywords:** oblique lateral interbody fusion, degenerative lumbar scoliosis, finite element analysis, osteoporosis, biomechanical stability

## Abstract

**Background:**

Few studies have been conducted on the biomechanical stability of oblique lumbar interbody fusion (OLIF) in conjunction with different fixation methods in patients with degenerative lumbar scoliosis (DLS) at varying bone densities. This study uses finite element analysis to assess the biomechanical stability of OLIF with various fixation techniques for treating DLS under differing bone densities.

**Methods:**

A three-dimensional finite element model of the lumbar spine (L1-S1) was created using CT scans from a Lenke-Silva IV DLS patient. The control group consisted of a posterior lumbar interbody fusion (PLIF) model. The experimental groups included OLIF Stand Alone (OLIF-SA), OLIF combined with unilateral pedicle screw fixation (UPSF), and OLIF combined with bilateral pedicle screw fixation (BPSF) models. Three bone density conditions—normal bone mass (NBM), osteopenia, and osteoporosis—were used to evaluate these models. The range of motion (ROM) of the surgical segment, the stress distribution of the Cage, endplate, and internal fixation, as well as the peak Von Mises stress, were evaluated by applying a vertical downward load of 400N and a torque of 7.5N·m in different directions.

**Results:**

Under different bone densities, compared to the PLIF model, the ROM of the surgical segment in the OLIF-SA model was significantly increased, whereas the ROM in the OLIF-UPSF and OLIF-BPSF models was similar to or lower than that of the PLIF. Under NBM and osteopenia, both OLIF-UPSF and OLIF-BPSF effectively reduced the peak Von Mises stress on the endplate and maintained surgical segment stability. However, under osteoporosis, the peak Von Mises stress on the endplate in the OLIF-UPSF model approached or exceeded the maximum yield stress of the endplate (60 MPa) in certain motion states, while OLIF-BPSF demonstrated superior biomechanical stability. Additionally, variations in bone density significantly affected the stress distribution of internal fixation devices, with more uniform stress observed in the OLIF-BPSF model under osteoporosis conditions.

**Conclusion:**

OLIF-BPSF may provide the best biomechanical stability for patients with DLS, especially osteoporosis patients. However, in patients with NBM and osteopenia, OLIF-UPSF remains an effective treatment option, which can ensure good biomechanical stability while obtaining significant minimally invasive advantages.

## 1 Introduction

Degenerative Lumbar Scoliosis (DLS) is a spinal deformity that develops in adulthood due to asymmetric degeneration of the intervertebral discs and facet joints. It is characterized by low back pain, radicular symptoms, and progressive deformity (Schwab et al., 2002; Aebi, 2005). When conservative treatments fail to alleviate the symptoms in DLS patients adequately, surgical intervention becomes a necessary therapeutic option (Kurra et al., 2018). Posterior lumbar interbody fusion (PLIF) has been widely employed for DLS treatment because it provides direct decompression and restores spinal balance. However, this procedure is associated with significant drawbacks, including substantial trauma, considerable blood loss, marked traction and interference with the spinal cord and nerve roots during surgery, and prolonged postoperative recovery (Mobbs et al., 2015).

Silvestre (Silvestre et al., 2012) introduced the Oblique Lumbar Interbody Fusion (OLIF) technique in 2012. As a minimally invasive spinal surgery approach, OLIF has demonstrated significant clinical advantages in the treatment of lumbar degenerative diseases over recent years (Kim et al., 2018; Yang et al., 2021; Zhang S. et al., 2022; Li et al., 2023). OLIF accesses the target intervertebral space through the natural anatomical corridor between the psoas major muscle and the abdominal aorta, thereby avoiding interference with the structures within the spinal canal. This technique facilitates the placement of larger-sized Cages to enhance mechanical support, effectively restoring the height of the intervertebral space and the physiological curvature of the lumbar spine. Additionally, OLIF offers significant minimally invasive advantages, including reduced surgical trauma, accelerated postoperative recovery, and a high fusion rate (Li et al., 2020; Gao et al., 2022; An et al., 2023). Although OLIF has demonstrated significant clinical advantages in the treatment of DLS, its biomechanical performance under osteoporosis remains inadequately evaluated. Wang (Wang et al., 2021) constructed a single-segment OLIF Stand Alone (OLIF-SA) model using finite element analysis to assess the biomechanical stability of OLIF-SA across varying bone density conditions. Their findings indicated that as bone density decreases, the maximum stress on the upper and lower endplates of the fusion segment significantly increases, thereby elevating the risk of Cage subsidence. Therefore, for patients with osteoporosis, OLIF-SA may not provide adequate stability. Moreover, DLS patients often present with complex lesions affecting multiple lumbar segments, and the prevalence of concurrent osteoporosis is notably high (Yuan et al., 2023). During OLIF surgery, DLS typically requires additional internal fixation to improve surgical outcomes and stability. Currently, bilateral pedicle screw fixation (BPSF) is widely recognized as the gold standard for the treatment of DLS, owing to its superior stability and high fusion rates (Wen et al., 2020). However, the combination of OLIF with BPSF results in a more complex surgical procedure, leading to extended operation times, increased anesthesia risks, and higher medical expenses for patients (Wen et al., 2020). In addition, Yang (Yang et al., 2021) reported that OLIF combined with unilateral pedicle screw fixation (UPSF) for the treatment of DLS can be used as an alternative fixation option, which reduces the operation time and medical costs while ensuring the therapeutic effect. However, existing studies are often limited by small sample sizes, and many of the patients involved have favorable bone conditions, which restricts the general applicability of the research findings. Currently, there is insufficient clinical data and objective biomechanical research to confirm the effectiveness of OLIF surgery combined with various fixation methods for DLS patients with differing bone densities.

Therefore, this study aims to evaluate the biomechanical stability of OLIF combined with different fixation methods for treating DLS under three different bone densities using finite element analysis. The surgical model of PLIF for DLS serves as the control, providing more accurate treatment guidance for clinical practice.

The remainder of this paper is organized as follows: Section 2 describes the development and validation process of the finite element models, including geometric reconstruction, material property assignment, and loading conditions. Section 3 presents a comparative analysis of the stability of the surgical models, along with an evaluation of the stress distribution and peak Von Mises stress in the endplates, Cage, and internal fixation. Section 4 critically evaluates the clinical implications of stress concentration phenomena, proposes a bone density-stratified fixation selection algorithm, and discusses technical limitations. Concluding remarks with future research directions are presented in Section 5.

## 2 Materials and methods

### 2.1 To construct and refine the DLS (L1-S1) model

This study selected the CT image data (slice thickness 0.625 mm) of a patient with Lenke-Silva IV DLS (female, 67 years old, weighing 63 kg, and standing 162 cm tall) from the Department of Orthopedics of the General Hospital of Ningxia Medical University. The DICOM format files were imported into MIMICS 20.0 (Materialise, Inc., Leuven, Belgium), and the images were processed by threshold segmentation to extract the complete three-dimensional model of L1-S1, which was then saved in STL format. Subsequently, the STL file was imported into Geomagic Studio 2021 (Geomagic, Inc., United States) for surface optimization and defect repair to generate the cortical bone model. Then, the cortical bone model was offset inward by 1 mm to obtain the cancellous bone model (Wang et al., 2021). After saving in STP file format successively, they were imported into SolidWorks 2018 (Dassault Systemes, France) for assembly. In the software, the annulus fibrosus, nucleus pulposus, endplate, and articular cartilage were drawn. The intervertebral disc consists of about 44% nucleus pulposus and 56% annulus fibrosus, with an endplate thickness of 1 mm (Iyer et al., 2010; Wang et al., 2022; Zhong et al., 2023). A complete three-dimensional model of the lumbar spine from L1 to S1 was ultimately created.

### 2.2 Construction of surgical models

**FIGURE 1:**
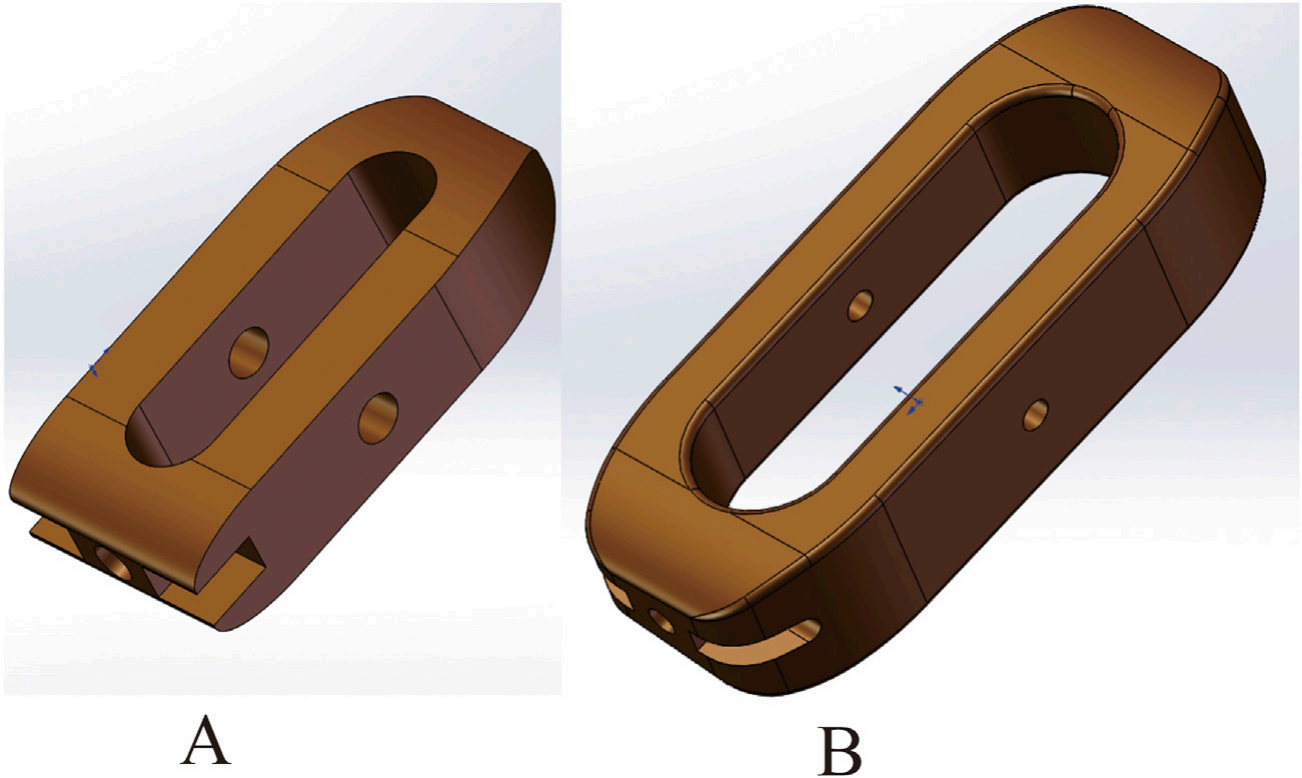
**(A)** Capstone cage, L/W/H: 26.0mm/10.0mm/10.0 mm. **(B)** Clydesdale cage, L/W/H:48mm/18mm/12 mm.

In this study, the Capstone Cage, which measures 26.0 mm in length, 10.0 mm in width, and 10.0 mm in height, was utilized for PLIF surgery. For OLIF surgery, the Clydesdale interbody fusion device was chosen, with dimensions of 48 mm in length, 18 mm in width, and 12 mm in height. The Clydesdale Cage features an arcuate design with a 6° lordotic angle between the superior and inferior surfaces. Both surfaces are contoured to enhance conformity with the vertebral endplates (Figure 1). To prevent potential stress singularity issues in subsequent analyses, the serrated surface of the fusion device was smoothed. The screws used have a diameter of 6.5 mm and a length of 45 mm, while the connecting rods have a diameter of 5.5 mm.

#### 2.2.1 PLIF model

In this study, the L2-3, L3-4, and L4-5 segments of the complete DLS (L1-S1) three-dimensional model were chosen as the surgical levels. A partial laminectomy was performed at the L2 and L5 vertebrae, while a total laminectomy was conducted at L3 and L4 to facilitate access to the surgical segments. The Capstone Cage was sequentially inserted at an oblique angle from the decompression side in the L2-3, L3-4, and L4-5 segments, involving partial removal of the annulus fibrosus and nucleus pulposus. The entry points for the pedicle screws were determined using the Weinstein method (Weinstein et al., 1988), followed by the bilateral placement of pedicle screws and the addition of connecting rods to construct the PLIF surgical model. The completed PLIF model was saved in the X. T format.

#### 2.2.2 OLIF-SA, OLIF-UPSF, and OLIF-BPSF models

Utilizing the three-dimensional DLS (L1-S1) model, the intervertebral discs at L2-3, L3-4, and L4-5 were segmented into four distinct regions based on their anterior and posterior edges. The discs and nucleus pulposus in regions two and three were excised, and Cages were inserted laterally, ensuring they adhered closely to the surface of the vertebral body. These Cages were then sequentially positioned on the left side of the L2-3, L3-4, and L4-5 segments to develop the OLIF-SA model. The Weinstein method (Weinstein et al., 1988) was employed to accurately identify the entry points for the placement of pedicle screws. Initially, unilateral pedicle screws were inserted, followed by the installation of a connecting rod, culminating in the construction of the OLIF-UPSF model. Subsequently, bilateral pedicle screws were placed using the same technique to create the OLIF-BPSF model. Finally, the OLIF-SA, OLIF-UPSF, and OLIF-BPSF models were sequentially saved as X. T format files.

### 2.3 Incorporate material properties, define ligaments, establish contact relationships, apply loads, and specify boundary conditions

**FIGURE 2:**
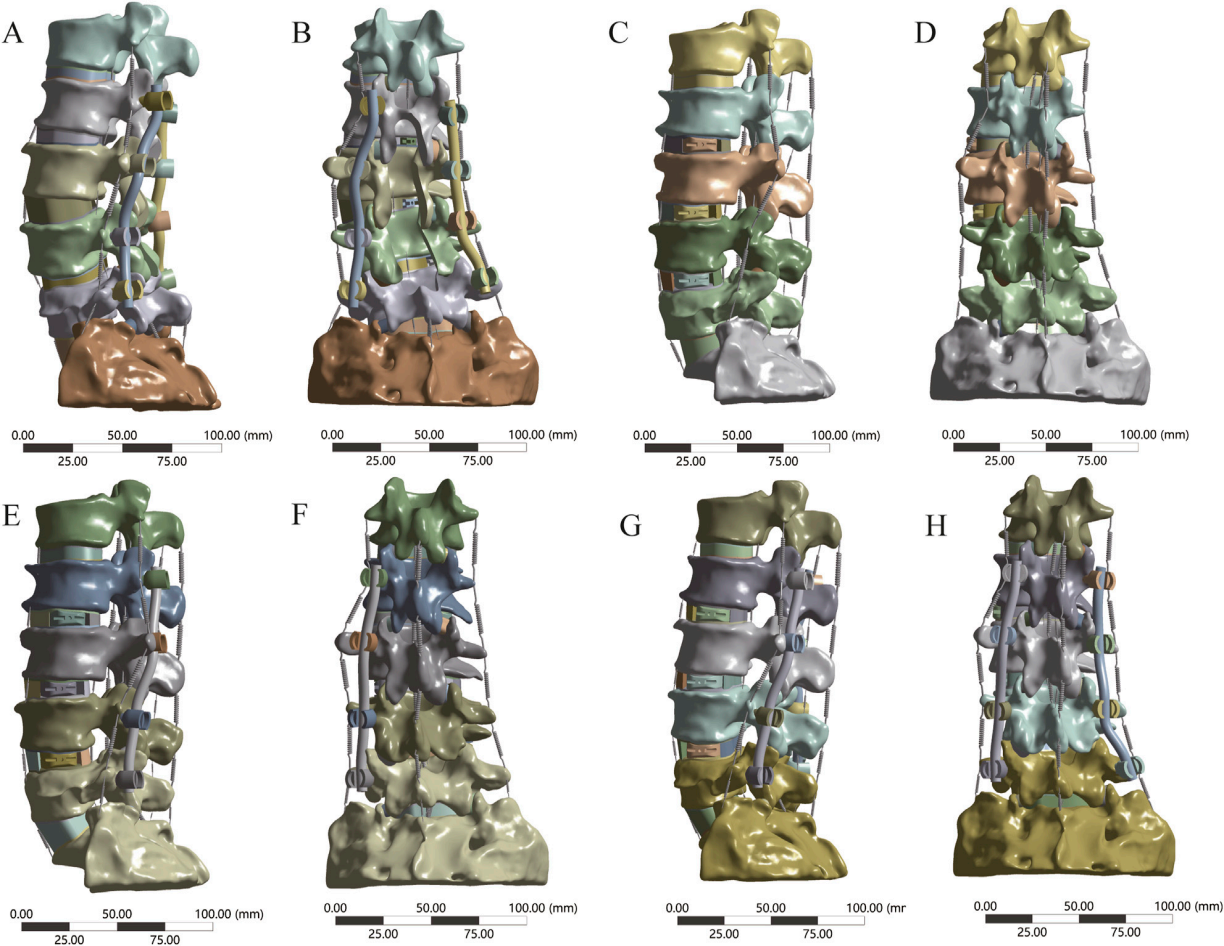
Four finite element models were developed in this study. **(A, B)** PLIF finite element model **(C, D)** OLIF-SA finite element model **(E, F)** OLIF-UPSF finite element model **(G, H)** OLIF-BPSF finite element model.

The PLIF, OLIF-SA, OLIF-UPSF, and OLIF-BPSF models were individually imported into ANSYS 18.0 (Ansys, Inc., United States) in X. T format files. A material library was established within ANSYS 18.0, and the material properties for each model were assigned accordingly (Wang et al., 2021; Fan et al., 2023; Kumaran et al., 2021). Compared to NBM, Young’s modulus of cortical bone and cancellous bone in the osteoporosis model decreased by 33% and 67%, respectively. Given that bone loss is characterized by a continuous T-value range, which finite element models cannot accurately simulate, the average Young’s modulus values of normal and osteoporotic bone were selected to represent varying degrees of bone mineral density reduction (Bereczki et al., 2021; Wang et al., 2021) (Table 1). Different Young’s Modulus were assigned to cortical and cancellous bone to sequentially establish the NBM model (PLIF 1, OLIF-SA 1, OLIF-UPSF 1, OLIF-BPSF 1), the osteopenic model (PLIF 2, OLIF-SA 2, OLIF-UPSF 2, OLIF-BPSF 2), and the osteoporosis (PLIF 3, OLIF-SA 3, OLIF-UPSF 3, OLIF-BPSF 3). The anterior longitudinal ligament, posterior longitudinal ligament, supraspinous ligament, interspinous ligament, ligamentum flavum, and intertransverse ligament were added sequentially to the corresponding anatomical positions in each model. (Huang et al., 2022; Fan et al., 2023). This process ultimately constructs complete finite element models for PLIF, OLIF-SA, OLIF-UPSF, and OLIF-BPSF (Figure 2). In the connection settings, the contact types between each model were defined as follows: the articular surface contact was set to “No separation,” and all other contact types were defined as “Boned.” Fine meshing was applied to all surgical models, with particular emphasis on ensuring high mesh quality in critical regions such as intervertebral discs and fusion interfaces to enhance analysis accuracy. Specifically, the mesh size for articular cartilage was set to 0.5 mm, while other regions were meshed at 1.5 mm. A uniform vertical downward load of 400 N was applied to the upper surface of the L1 vertebral body, while different directional torques of 7.5 N·m were simultaneously applied to simulate six physiological states: flexion, extension, left flexion, right flexion, left rotation, and right rotation. This experimental setup was used to investigate the range of motion (ROM) of the lumbar spine model under these six conditions, as well as the stress distribution in the fusion Cage, surgical segment endplates, and internal fixation devices, including the peak Von Mises stress.

**TABLE 1:** Properties of each material in finite element models.

Material	Young’s modulus (MPa)	Poisson’s ratio	Stiffness (N/mm)	References
Cortical bone (NBM)	12,000	0.3	-	Bereczki et al., (2021), Wang et al. (2021)
Cortical bone (Osteopenia)	10,020	0.3	-	Wang et al. (2021)
Cortical bone (Osteoporosis)	8,040	0.3	-	Bereczki et al., (2021), Wang et al. (2021)
Cancellous bone (NBM)	100	0.2	-	Bereczki et al., (2021), Wang et al. (2021)
Cancellous bone (Osteopenia)	67	0.2	-	Wang et al. (2021)
Cancellous bone (Osteoporosis)	34	0.2	-	Bereczki et al., (2021), Wang et al. (2021)
Endplate	2000	0.2	-	Fan et al., (2023) Wang et al. (2021)
Anulus fibrosus	4.2	0.45	-	Fan et al., (2023), Wang et al. (2021)
Nucleus pulposus	1	0.499	-	Fan et al., (2023), Wang et al. (2021)
Articular cartilage	25	0.25	-	Fan et al., (2023), Wang et al. (2021)
Ligaments				
Anterior longitudinal ligament	7.8	-	8.74	Kumaran et al. (2021)
Posterior longitudinal ligament	10	-	5.83	Kumaran et al. (2021)
Supraspinous ligament	8	-	15.38	Kumaran et al. (2021)
Interspinous ligament	10	-	0.19	Kumaran et al. (2021)
ligamentum flavum	15	-	15.75	Kumaran et al. (2021)
Intertransverse ligament	10	-	2.39	Kumaran et al. (2021)
Cage (PEEK)	3,600	0.3	-	Bereczki et al., (2021), Wang et al. (2021)
Internal fixation (Ti-6A1-4V)	11,000	0.3	-	Bereczki et al. (2021)

## 3 Results

### 3.1 Model validation

**FIGURE 3:**
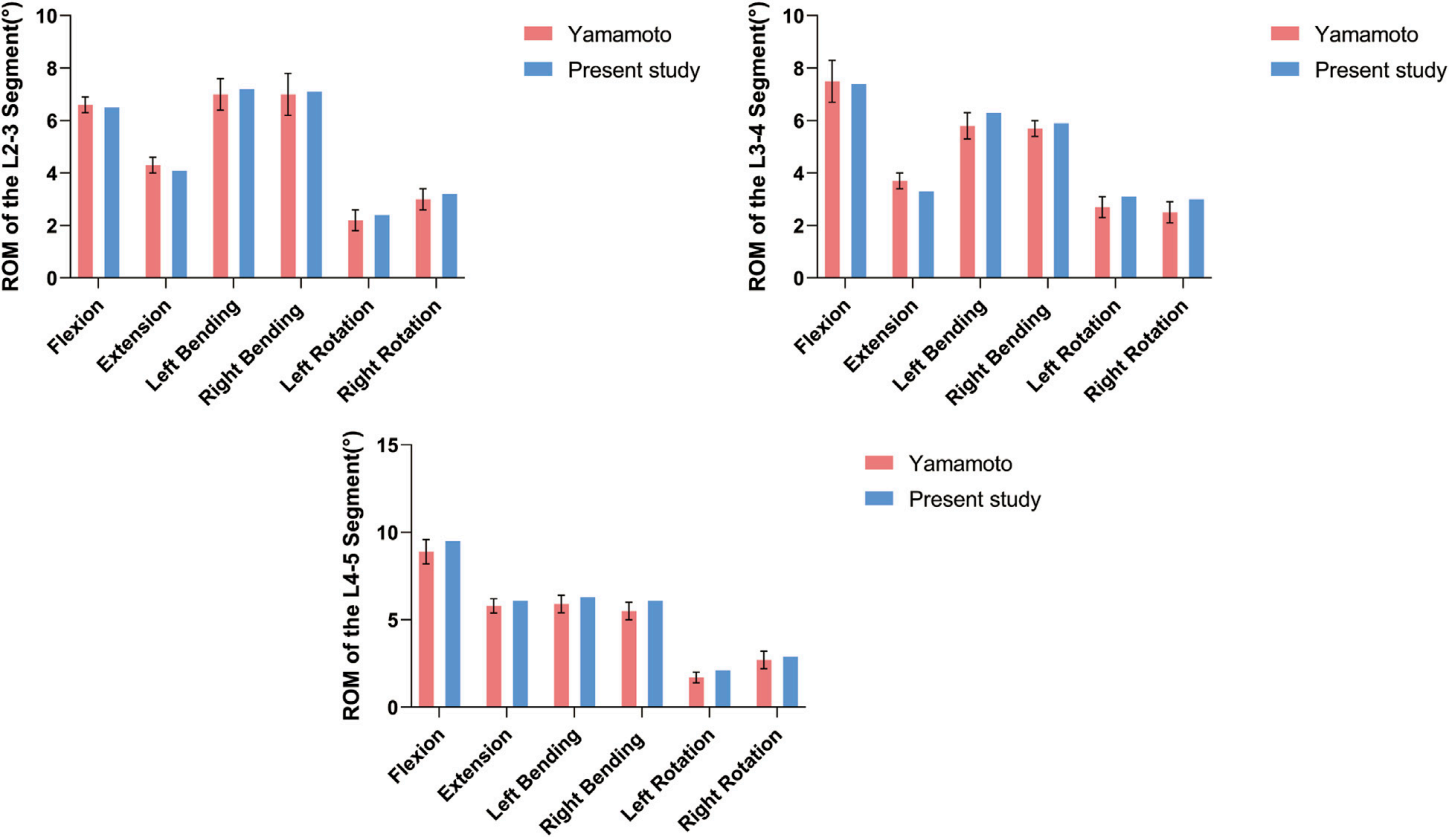
DLS finite element modeling of surgical segment ROM compared with the cadaveric study of Yamamoto et al. at different states of motion.

Before conducting the finite element analysis, we validated the three-dimensional finite element model of DLS (L1-S1). The validation was performed by comparing our results with those of Yamamoto (Yamamoto et al., 1989) under identical loading and boundary conditions. It was observed that the ROM of the L2-5 segment in each motion state of our model closely corresponded to their experimental findings (Figure 3; Table 2). Consequently, this DLS (L1-S1) three-dimensional finite element model is deemed appropriate for subsequent biomechanical analyses.

**TABLE 2:** Comparison of the present study of surgical segment ROM with the cadaveric study of Yamamoto et al. in different states of motion.

Motion states	L2-3		L3-4		L4-5	
	Yamamoto et al.	Present study	Yamamoto et al.	Present study	Yamamoto et al.	Present study
Flexion	6.6 ± 0.3	6.5	7.5 ± 0.8	7.4	8.9 ± 0.7	9.5
Extension	4.3 ± 0.3	4.1	3.7 ± 0.3	3.3	5.8 ± 0.4	6.1
Left Bending	7 ± 0.6	7.2	5.8 ± 0.5	6.3	5.9 ± 0.5	6.3
Right Bending	7 ± 0.8	7.1	5.7 ± 0.3	5.9	5.5 ± 0.5	6.1
Left Rotation	2.2 ± 0.4	2.4	2.7 ± 0.4	3.1	1.7 ± 0.3	2.1
Right Rotation	3 ± 0.4	3.2	2.5 ± 0.4	3	2.7 ± 0.5	2.9

### 3.2 ROM

**FIGURE 4:**
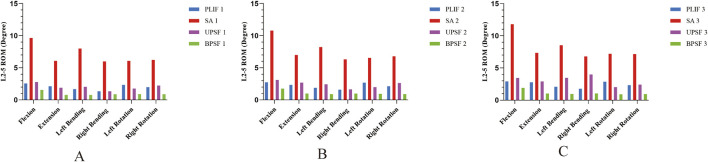
ROM of surgical segments at different bone densities for all surgical models. **(A)** NBM Model. **(B)** Osteopenia Model. **(C)** Osteoporosis Model.

To comprehensively evaluate the stability of four surgical models under three bone densities, this study applied a vertical downward load of 400 N and torques of 7.5 N·m in different directions to all surgical models and compared the ROM of the surgical segments. Under different bone densities, compared with the control group PLIF model, the ROM of the OLIF-SA surgical segment significantly increased in six directions: flexion, extension, left flexion, right flexion, left rotation, and right rotation; the ROM of the OLIF-UPSF model was similar to that of the PLIF model; the ROM of the OLIF-BPSF model was the lowest among all models. Under all motion states, the ROM of the four osteoporosis surgical models was the largest, that of the osteopenia model was in the middle, and that of the NBM model was the smallest (Figure 4).

### 3.3 Stress distribution and peak Von Mises stress in cage

**FIGURE 5:**
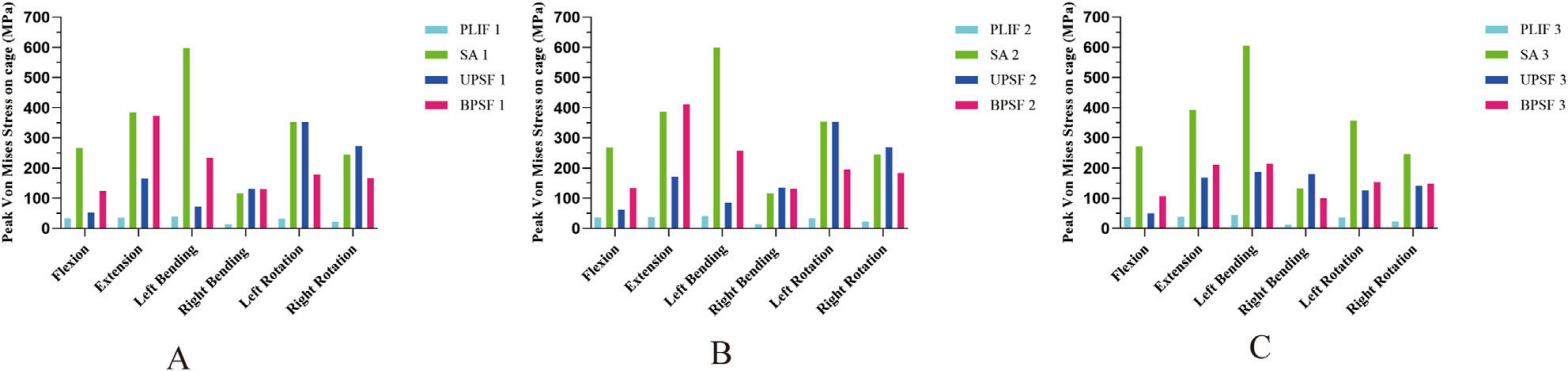
Peak Von Mises stress of Cage at different bone densities for all surgical models. **(A)** NBM Model. **(B)** Osteopenia Model. **(C)** Osteoporosis Model.

In the NBM model, the PLIF model had the lowest peak Von Mises stress of the Cage in all motion states; the OLIF-SA model had the largest increase in the peak Von Mises stress of the Cage in all motion states, especially in left flexion, where the peak Von Mises stress of the Cage reached 597.29 MPa. Compared with the OLIF-SA model, the OLIF-UPSF model significantly reduced the peak Von Mises stress of the Cage in flexion, extension, left flexion, and right flexion; while the OLIF-BPSF model significantly reduced the peak Von Mises stress of the Cage in left rotation and right rotation (Figure 5A). With the decrease in bone density, under osteopenia (Figure 5B) and osteoporosis (Figure 5C), the peak Von Mises stress in the Cage of all surgical models was higher than that in the NBM model. The trend remained consistent with that observed in the NBM model: the PLIF model exhibited the lowest peak Von Mises stress, while the OLIF-SA model showed the highest peak Von Mises stress.

### 3.4 Stress distribution and peak Von Mises stress in endplates of surgical segments

**FIGURE 6:**
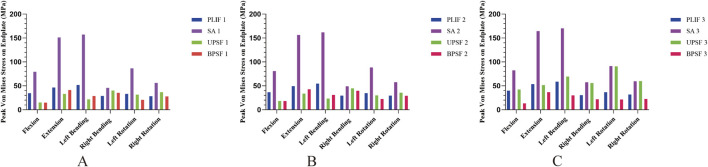
Peak Von Mises stresses in the endplates of all surgical models at different bone densities. **(A)** NBM Model. **(B)** Osteopenia Model. **(C)** Osteoporosis Model.

**FIGURE 7:**
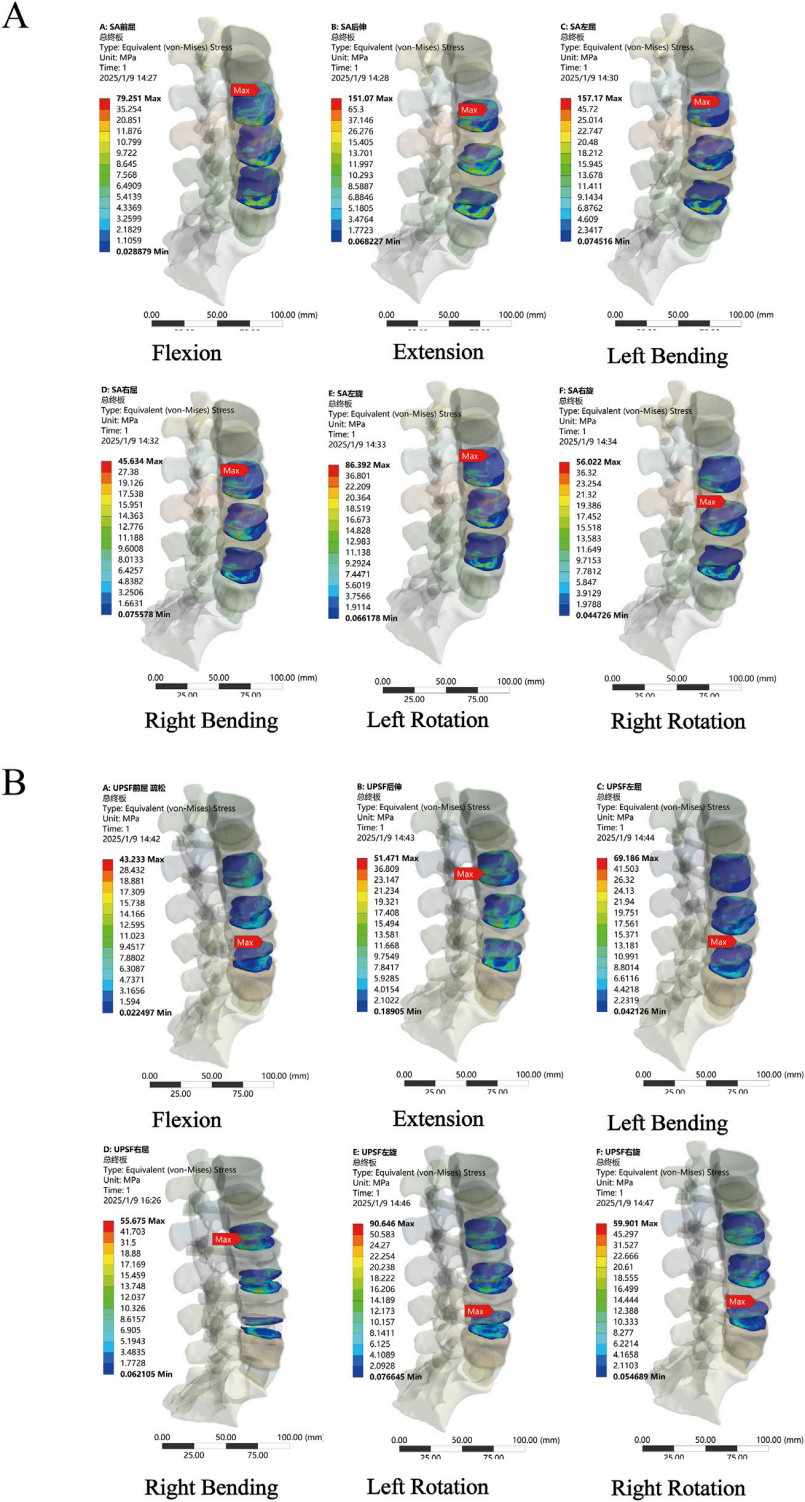
Stress distribution of surgical segment endplates. **(A)** OLIF-SA NBM model **(B)** OLIF-UPSF osteoporosis model.

In the NBM model (Figure 6A), the peak Von Mises stresses in the endplates of the surgical segments for the PLIF model were as follows: 34.396 MPa in anterior flexion, 46.411 MPa in posterior extension, 51.665 MPa in left lateral bending, 28.828 MPa in right lateral bending, 33.01 MPa in left rotation, and 28.261 MPa in right rotation. In comparison, the OLIF-SA model exhibited significantly higher peak Von Mises stresses across all six kinematic states: 79.251 MPa in anterior flexion, 151.07 MPa in posterior extension, 157.17 MPa in left lateral bending, 45.634 MPa in right lateral bending, 86.392 MPa in left rotation, and 56.022 MPa in right rotation. Notably, all these values exceeded the maximum yield stress of the endplate (60 MPa) (Figure 7A). In the OLIF-UPSF model, the peak Von Mises stress of the endplate at the surgical segment was significantly reduced in all motion states except right flexion (40.103 MPa) and right rotation (36.681 MPa), with reductions ranging from 5% to 58%. For the OLIF-BPSF model, the peak Von Mises stress of the endplate at the surgical segment was higher than that of the PLIF model only in right flexion (35.279 MPa), while it was significantly reduced in other motion states, with reductions ranging from 2% to 57%.

In the osteopenia model (Figure 6B), the peak Von Mises stress of the endplates at the surgical segments was higher in all four surgical models compared to the NBM model. Except for the OLIF-SA model, the peak Von Mises stress in the other three surgical models did not exceed the maximum yield stress of the endplate. Compared with the PLIF model, the changes in the peak Von Mises stress of the endplates at the surgical segments in the OLIF-SA, OLIF-UPSF, and OLIF-BPSF models exhibited a trend similar to that observed in the NBM model.

In the osteoporosis model (Figure 6C), the peak Von Mises stress of the endplates at the surgical segments in all surgical models was higher than that observed in the NBM model. Specifically, in the OLIF-UPSF model, the peak Von Mises stress of the endplate at the surgical segment approached or exceeded the maximum yield stress under left flexion (69.186 MPa), left rotation (90.646 MPa), and right rotation (59.901 MPa) (Figure 7B). For the OLIF-BPSF model, the peak Von Mises stresses of the endplate at the surgical segment under six motion states were as follows: flexion 13.179 MPa, extension 36.804 MPa, left flexion 30.036 MPa, right flexion 21.964 MPa, left rotation 21.493 MPa, and right rotation 22.662 MPa.

### 3.5 Stress distribution and peak Von Mises stress in internal fixation

**FIGURE 8:**
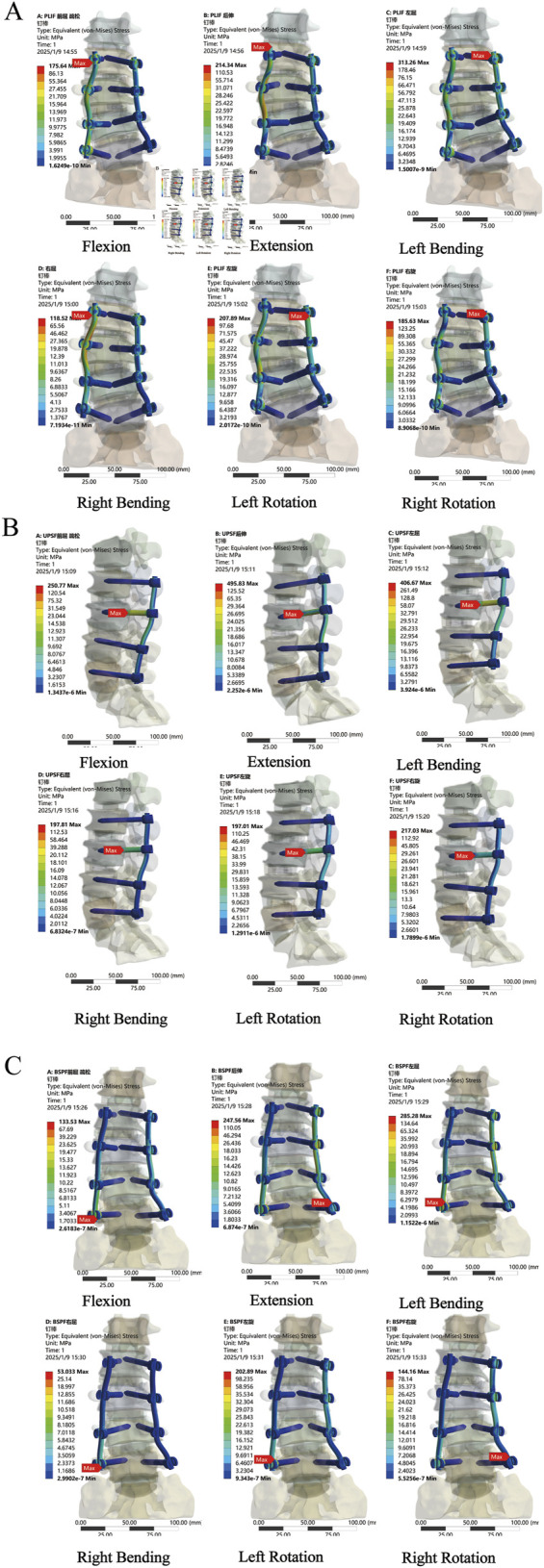
Stress distribution analysis of internal fixation in osteoporosis across various surgical models. **(A)** PLIF model **(B)** OLIF-UPSF model **(C)** OLIF-BPSF model.

**FIGURE 9:**
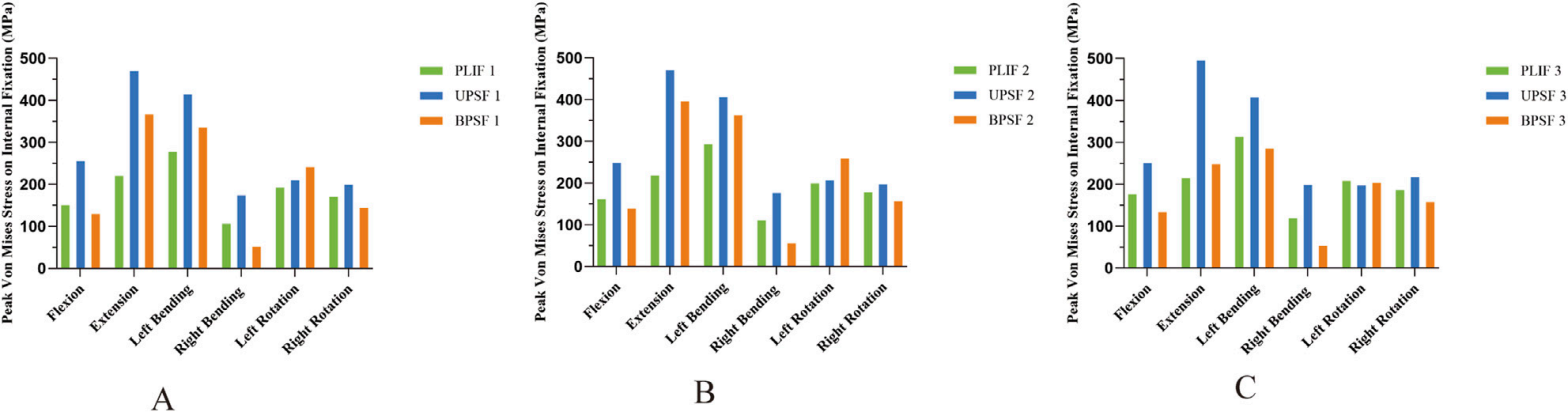
Peak Von Mises stresses for internal fixation across diverse bone densities in various surgical models. **(A)** Normal Model. **(B)** Osteopenia Model. **(C)** Osteoporosis Model.

Compared with the PLIF model, the peak Von Mises stress of the OLIF-UPSF internal fixation device increased across all motion states. Specifically, the increases were as follows: 9%–114% in the NBM model, 4%–117% in the bone density reduction model, and −5%–131% in the osteoporosis model. Notably, the maximum increase was consistently observed in the extension state. In the osteoporosis model, the peak Von Mises stress value for the OLIF-UPSF device reached 495.83 MPa at the junction between the L3 pedicle screw and the vertebral body. Under NBM and osteopenia, compared with the PLIF model, the peak Von Mises stress of the OLIF-BPSF internal fixation device increased in extension, left flexion, and left rotation. Specifically, the increases were 21%–67% in the NBM model and 24%–82% in the osteopenia model, with the maximum increase consistently observed in the extension state. In contrast, under osteoporosis, except for the extension state, the peak Von Mises stress of the OLIF-BPSF internal fixation decreased in other movement states, with a reduction range of 2%–55%, and the maximum reduction occurred in the right flexion state. (Figures 8, 9).

## 4 Discussion

With the intensification of population aging, DLS has emerged as a significant spinal condition affecting the health of middle-aged and elderly individuals (Aebi, 2005). DLS, characterized by its complex pathophysiology and therapeutic challenges, continues to be a focal point in spinal surgery research (Qiu et al., 2022). Given that DLS patients are typically older and often suffer from osteopenia and multiple comorbidities, traditional PLIF surgery is associated with substantial trauma, high intraoperative blood loss, prolonged postoperative bed rest, and an elevated risk of complications (Liu et al., 2023). Consequently, minimally invasive surgical techniques have emerged as a new option for treating DLS. There remains controversy regarding whether OLIF can effectively treat DLS through decompression and spinal stabilization, as well as the optimal choice of internal fixation (Yang et al., 2021). For patients with DLS, selection should be guided by factors such as bone density, degree of deformity, and comorbidities (Bereczki et al., 2021; Yang et al., 2021; Zhang Y. et al., 2022).

This study systematically evaluated the biomechanical stability and stress distribution of OLIF combined with different fixation methods (OLIF-SA, OLIF-UPSF, OLIF-BPSF) compared to PLIF in treating DLS under three distinct bone densities using finite element analysis. The results demonstrated that, across varying bone densities, the ROM of the OLIF-SA surgical segment significantly increased, while the ROM of the OLIF-UPSF and OLIF-BPSF segments was comparable to or lower than that of PLIF. This suggests that under NBM, OLIF-SA exhibits increased ROM due to insufficient stability, whereas OLIF-UPSF and OLIF-BPSF maintain or reduce ROM, achieving stability similar to the PLIF model. As bone density decreases, the ROM of all surgical models progressively increases, likely due to osteoporosis-induced bone fragility and reduced support. These findings are consistent with those reported by Song et al. (2021), who found that OLIF-BPSF had the smallest ROM across all motion states, while OLIF-SA had the largest ROM. Additionally, under the same fixation method, osteoporosis increases the ROM of the surgical segment, but OLIF-BPSF still provides relatively stable biomechanical performance.

Previous clinical studies have reported that the subsidence rate of Cages following OLIF surgery ranges from 7.2% to 46.7% (Shen et al., 2023). The integrity of the endplate is essential for maintaining the load-bearing capacity of the vertebral body and the stability of the Cage. Excessive stress on the endplate can lead to damage and subsequently increase the risk of Cage subsidence (Yu et al., 2024). In models with NBM, the peak Von Mises stress of the Cage in the PLIF model was the lowest, whereas the peak Von Mises stress increase in the OLIF-SA model was the most pronounced. This phenomenon may be attributed to the differences in Cage dimensions between the two models: the Cage in the OLIF-SA model is designed to be larger, thereby bearing a greater proportion of the stress distribution within the intervertebral space. As bone density decreases, the peak Von Mises stress of the Cage in all surgical models significantly increases, further corroborating the adverse impact of osteoporosis on the stability of the OLIF-SA model. In this study, under NBM, the peak Von Mises stress of the endplate at the surgical segment of the OLIF-SA model exceeded the maximum yield stress of the endplate (60 MPa) in all six motion states (79.251 MPa, 151.07 MPa, 157.17 MPa, 45.634 MPa, 86.392 MPa, and 56.022 MPa, respectively) (Wang et al., 2021). In contrast, the OLIF-BPSF model exhibited superior biomechanical stability across all bone density conditions, particularly in osteoporosis, where the internal fixation stress distribution was more uniform, thereby significantly reducing the risk of Cage subsidence and endplate fractures. For patients undergoing OLIF treatment, the risk of endplate fractures and Cage subsidence is markedly elevated in any motion state. Consequently, internal fixation must be employed as an adjunct to OLIF-SA treatment for such patients. Liu et al. (2022) constructed a three-segment (L2-5) lumbar lateral interbody fusion (LLIF) surgical model combined with four fixation methods and found that the peak endplate stress in the Stand-Alone model significantly increased, exceeding that of the additional fixation (BPSF) model by 133.6%, 175.1%, and 90.7% in flexion, extension, lateral bending, and axial rotation, respectively.

BPSF is considered the gold standard for treating DLS, known for its high stability and fusion rate (Zhang et al., 2022b). The results of this study reinforce the idea that, particularly under osteoporosis, the OLIF-BPSF model demonstrates superior biomechanical stability. However, the complexity of the OLIF-BPSF fixation procedure prolongs the operation time, increases the risk associated with anesthesia, and raises medical expenses for patients. Consequently, there is an urgent need to identify a fixation method that can provide adequate stability while minimizing surgical trauma and costs. Yang (Yang et al., 2021) demonstrated that, without compromising therapeutic outcomes, OLIF-UPSF can enhance surgical efficiency and reduce the economic burden on patients. In this study, both OLIF-UPSF and OLIF-BPSF effectively reduced peak endplate stress and maintained segmental stability under conditions of NBM and osteopenia. However, under osteoporosis, the peak Von Mises stress of the endplate in the OLIF-UPSF approached or exceeded the maximum yield stress of the endplate (60 MPa) during left flexion and left and right rotation (69.186 MPa, 90.646 MPa, and 59.901 MPa, respectively), indicating that OLIF-UPSF may not be an effective treatment option for patients with DLS and osteoporosis.

Moreover, this study revealed that alterations in bone density have differing impacts on the biomechanical performance of OLIF when assessed alongside various fixation methodologies. Compared with the PLIF model, the peak Von Mises stress of internal fixation in the OLIF-UPSF model increased across three different bone densities in various motion states, particularly during extension (the maximum stress reached 495.83 MPa under osteoporosis). Although the peak Von Mises stress of OLIF-UPSF in the osteoporosis model during extension was lower than the maximum yield strength of titanium alloy internal fixation (795–827 MPa), it approached the fatigue strength (500 MPa) (Puttlitz et al., 2001; Huang et al., 2022). Fatigue strength refers to the maximum stress that a material can withstand under repeated cyclic loading, which differs from the stress limit observed under a single static load (Huang et al., 2022). Consequently, in patients with osteoporosis, OLIF-UPSF may be susceptible to internal fixation fractures due to prolonged stress concentration, potentially compromising the long-term clinical outcomes of the surgery. In contrast, under osteoporosis, the OLIF-BPSF model exhibits significantly lower peak Von Mises stress in the internal fixation compared to PLIF across all motion states except extension, with reductions ranging from 2% to 55%, the most pronounced reduction being in right lateral flexion. This phenomenon can be attributed to the preservation of the complete posterior ligamentous complex in the OLIF-BPSF model, which more effectively disperses stress and reduces stress concentration on the internal fixation device, thereby lowering the risk of loosening or fracture. As bone quality changes, the stiffness and stability at the interface between the vertebral body and the screw will also exhibit variation (Chen et al., 2024). Under NBM and osteopenia, the bone remains relatively hard, resulting in a larger contact area between the screw and the bone and enhanced stability. Consequently, the stress distribution is more uniform. In contrast, under osteoporosis, the strength and stiffness of the bone significantly decrease, leading to a reduced contact area between the screw and the bone, diminished stability, and increased stress concentration on the screw. Additionally, osteoporosis reduces Young’s modulus and compressive strength of the vertebral body, which may increase relative displacement between the vertebral body and the fixation system in the same radial direction, potentially causing loosening or fracture of the internal fixation device (Liu et al., 2022). Although OLIF-UPSF exhibits slightly lower biomechanical stability compared to OLIF-BPSF, it still holds significant clinical application value (Wen et al., 2020). Therefore, for DLS patients with NBM or osteopenia, the use of OLIF-UPSF can ensure adequate biomechanical stability while providing notable minimally invasive advantages.

This study has several limitations. Firstly, during the model construction process, simplifications were made to the anatomical structure and material properties, such as removing the serrated structures of the Cage and screws and simplifying the complexity of soft tissues like ligaments. The simplified treatment of the intervertebral disc (e.g., ignoring the multilayered structure of the annulus fibrosus and the viscoelasticity of the nucleus pulposus) as well as the omission of complex mechanisms of degeneration, such as nutrient supply, cellular metabolism, external factors (e.g., cigarette smoking), age-related changes, and mechanical factors may lead to limitations in the accuracy of the model in simulating the true biomechanical behavior of the intervertebral disc (Volz et al., 2021). These simplifications may introduce deviations in the model’s ability to accurately simulate actual biomechanical behavior, thereby limiting its capacity to fully reflect the complex mechanical characteristics of the human spine. Secondly, the reduction of bone density and osteoporosis was simulated by reducing the Young’s modulus of the bone. However, these conditions are complex pathological processes involving reductions in bone density, changes in bone microstructure, and alterations in bone geometry (Al-Barghouthi et al., 2020). The model did not fully account for these factors, which may impact the accuracy of the research findings. Thirdly, this study only simulated basic movement patterns of the human body, whereas the actual movement of the human spine is more intricate and can involve combinations of multiple movement states. Consequently, it failed to comprehensively evaluate the biomechanical stability of different fixation methods under complex movement conditions. Fourthly, This study was based on a single spine model, which could not adequately consider the diversity of anatomical structures, bone conditions, and pathological changes among different individuals, thus limiting the generalizability of the findings. Finally, the finite element analysis primarily focused on immediate post-surgical biomechanical stability and lacked an assessment of long-term clinical outcomes. Given the influence of natural degeneration and other factors, our results can only reflect a trend. Therefore, it is recommended to compare these findings with *in vitro* experimental results to gain a more comprehensive understanding.

## 5 Conclusion

In conclusion, OLIF-BPSF may provide superior biomechanical stability for patients with DLS, particularly those with osteoporosis. However, given the minimally invasive nature and cost-effectiveness of the procedure, OLIF-UPSF remains a viable treatment option for DLS patients with NBM or osteopenia.

## Data Availability

The raw data supporting the conclusions of this article will be made available by the authors, without undue reservation.
